# The amplitude of low frequency fluctuation and spontaneous brain activity alterations in age-related macular degeneration

**DOI:** 10.3389/fmed.2024.1507971

**Published:** 2025-01-22

**Authors:** Yan Yan Zhang, Jin Yu Hu, Qian Ling, San Hua Xu, Min Kang, Hong Wei, Jie Zou, Quanyong Yi, Gang Tan, Yi Shao

**Affiliations:** ^1^Ningbo Eye Hospital, Wenzhou Medical University, Ningbo, China; ^2^Department of Ophthalmology, The First Affiliated Hospital of Nanchang University, Jiangxi Centre of National Clinical Research Ophthalmology Center, Nanchang, China; ^3^Department of Ophthalmology, The First Affiliated Hospital of University of South China, Hunan Center of National Ocular Disease Clinical Research Center, Hengyan, China; ^4^Department of Ophthalmology, Shanghai General Hospital, Shanghai Jiao Tong University School of Medicine, National Clinical Research Center for Eye Diseases, Shanghai, China

**Keywords:** the amplitude of low-frequency fluctuation, age-related macular degeneration, spontaneous brain activity, functional magnetic resonance imaging, resting brain activity

## Abstract

**Background:**

Wet age-related macular degeneration (wAMD) is a vision-threatening eye disease worldwide. The amplitude of low-frequency fluctuation (ALFF) method was used to observe changes in spontaneous brain activity, which may help to investigate the underlying pathological mechanism of AMD.

**Methods:**

Eighteen patients with wAMD and 18 age- and gender-matched healthy controls (HCs) were recruited. The ALFF method was used on each subject and mean ALFF values were compared between groups. The receiver operating characteristic (ROC) curve was used to compare the two groups.

**Results:**

ALFF values in the temporal lobe and limbic lobe/parahippocampal gyrus were significantly higher than controls, while values in the postcentral gyrus were significantly lower. The under the curve of the ROC (AUC) of the three regions shows high accuracy of the diagnosis.

**Conclusion:**

The abnormal spontaneous brain activity of patients with AMD suggests scope for the use of ALFF in the diagnosis or prognosis in AMD.

## 1 Introduction

The role of the visual system is to process visual information via neural pathways connecting the eyes, optic nerve, lateral geniculate body, and visual cortex (1). Impairment of this system is a serious health problem. In recent years, a range of eye diseases, such as myopia, optic neuritis, glaucoma, and wet age-related macular degeneration (wAMD) are increasingly common. AMD, as the name suggests, is related to age, and features painless, progressive degeneration of the macular. This region is responsible for visual acuity, so the disease will seriously affect the daily life of patients, and is a very harmful disease. Globally, it is a major cause of irreversible blindness in the elderly (2). With an increase in the older population in many countries, more than 20% people may suffer from this disease (3). In China, although AMD is not the main cause of blindness, the incidence has increased in recent years. One study showed that in developed cities, the incidence of AMD has reached 15.5% (4). Regarding the cause of AMD, we know that there are many risk factors for the disease, however, the pathophysiological mechanism of AMD has not been completely elucidated (5). It is believed that it is the result of a combination of metabolism, genetics, environment, and other factors (6). Abnormalities of photoreceptor cells, retinal pigment epithelium, and Bruch’s membrane/choroid complex usually progress to cause geographic atrophy or neovascularization (7). AMD is categorized as wet or dry according to the pathological stage. wAMD is characterized by neovascularization and is the most common form.

**Figure 1:**
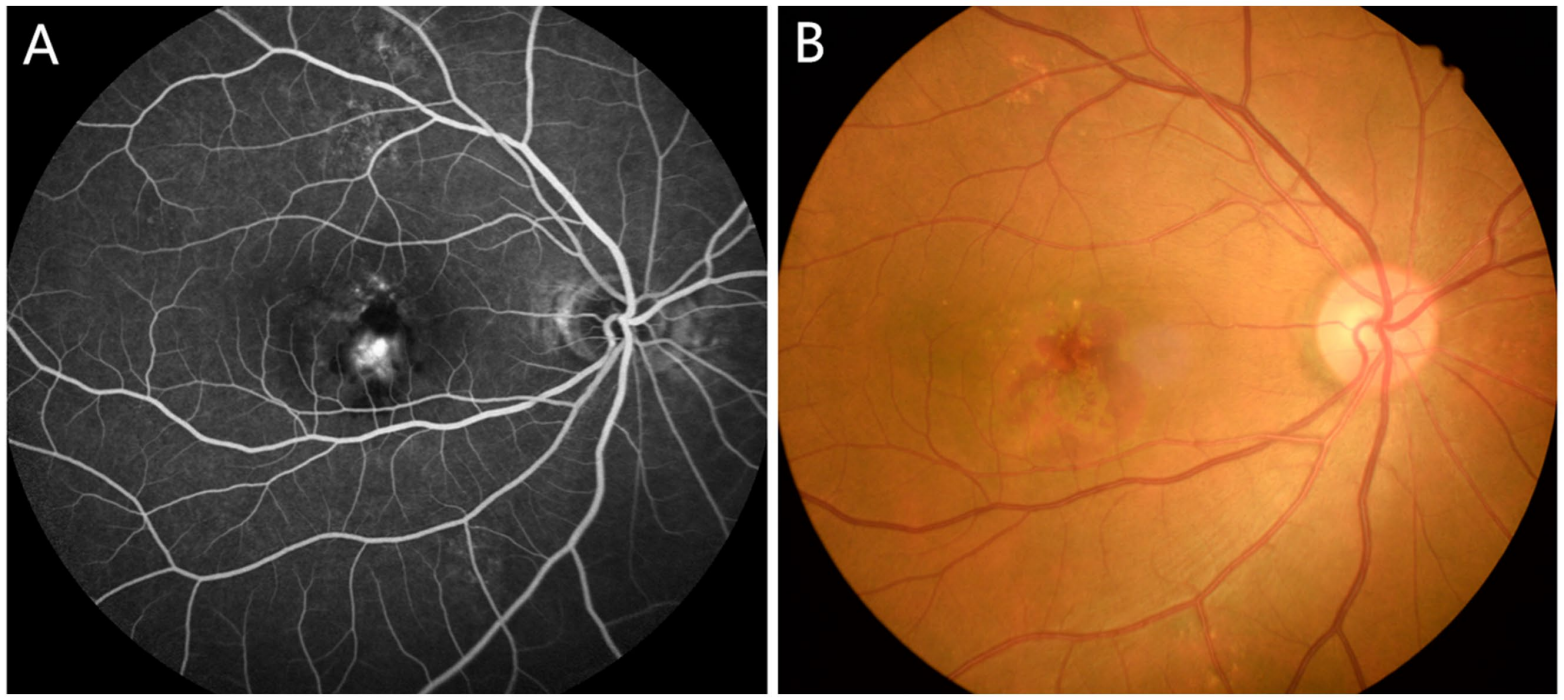
Typical fundus photographs of AMD patients. **(A)** Funduscopy in AMD patients; **(B)** Fluorescein angiography in AMD patients. AMD, age-related macular degeneration.

The diagnosis of AMD is aided by examination methods such as fundus angiography, optical coherence tomography (OCT), fundus autofluorescence, and confocal laser fundus imaging. Clinical findings allow classification based on published consensus as follows (8): (1) A lack of visible drusen or abnormal pigmentation indicates no sign of AMD; (2) The presence of small drusen (<63 μm), also known as Drupelet, is considered a normal aging change without risk of progression to AMD; (3) Moderate drusen (63 ~ 125 μm) with no pigment abnormalities indicates early AMD; (4) Large drusen or a combination of moderate drusen with pigment abnormalities indicates moderate AMD; (5) Neovascular AMD or geographic atrophy should be deemed advanced AMD. The fundus of a patient with AMD is shown in Figure 1.

AMD is a disease affecting central vision, and therefore reducing the ability to drive, read, and recognize faces (9). At present, the disease cannot be cured clinically, and the current treatment method is intravitreal injection (10). However, it is understood that AMD has different pathological types, so the treatment methods also vary. Central vision is significantly affected in the late stage of GA, and most patients with dry AMD are already in the late stage. At this time, treatment for AMD is mainly based on a number of factors including the inhibition of inflammation and nutrient nerves, Nutrient nerve is a treatment method that protects photoreceptors by providing drugs. wAMD is characterized by the formation of choroidal neovascularization (CNV) and can cause subretinal exudation and bleeding. Therefore, current treatment methods for this form of the disease are mainly laser photocoagulation, photodynamic therapy, drugs, and surgery. Anti-VEGF medication is one of the standard treatments for neovascular AMD (11–13). A large body of research exists on the pathogenesis, treatment, diagnosis, and other aspects of AMD, but relatively little on the changes in brain function related to AMD. Previous studies have confirmed that brain function changes are associated with many fundus diseases, such as retinal detachment, diabetic retinopathy, hypertensive retinopathy and so on (14–16). Therefore, this study investigates brain function of patients with AMD compared with healthy controls.

The amplitude of low-frequency fluctuation (ALFF) was used to explore its potential as a tool for early diagnosis of AMD. The technology of functional magnetic resonance imaging (fMRI) offers a means by which anomalies in brain function can be assessed and allows increased understanding of pathology of the central nervous system (17–19). Resting state functional magnetic resonance imaging (rs-fMRI) has been widely applied to monitor human brain function (20). The ALFF approach is a reliable and reproducible rs-fMRI method to evaluate the spontaneous fluctuations of resting brain activity (21–28).

At present, the ALFF method is widely used in the study of eye diseases, such as blindness,1 hypertensive retinopathy (15), strabismic amblyopia (29), unilateral acute open globe injury (30), acute eye pain (31), and corneal ulcer (32). In the present study, it is used to assess brain function in patients with AMD compared with healthy controls with the aim of better understanding the pathogenesis of AMD and to explore whether this new technology can enhance early diagnosis.

## 2 Methods

### 2.1 Patients

Eighteen patients with AMD (8 females and 10 males) were recruited from the Department of Ophthalmology at The First Affiliated Hospital of Nanchang University (33). These subjects met the following criteria: (1) Disease onset is related to age; (2) Fundus photographs show disease-related lesions at the macula; (3) Drusen size and quantity of abnormal pigment exceeds pre-determined minimum criteria; (4) The patient has no other eye disease; (5) The patient has no brain disease with the potential to affect brain function. Patients with mental illness or with unhealthy lifestyle factors such as alcoholism or smoking were excluded.

The two groups were similar in terms of gender, age, and weight. Control subjects were included if they satisfied the following criteria: (1) no functional abnormalities found in a head MRI (e.g., cerebral hemorrhage or cerebrovascular disease); (2) no MRI contraindications (no mental in the body, no high fever, and pregnant women with younger gestational weeks); (3) There are no risk factors for AMD, such as advanced age and smoking.

The research was approved by the Human Research Ethics Committee of the First Affiliated Hospital of Nanchang University. Each participant understood the aim, technology and possible risks of the research, and signed an informed consent agreement.

### 2.2 MRI data collection

The Trio 3-Tesla MR scanner (Siemens, Munich, Germany) was used. Before scanning, participants were asked to relax, close their eyes, and minimize movement (30). To obtain functional data, a 3D metamorphic gradient echo pulse sequence was used. The following parameters were used for a 176 image scan: acquisition matrix 256 × 256; field of view 250 × 250 mm; echo time 2.26 ms; repetition time 1,900 ms; thickness 1.0 mm; gap 0.5 mm; flip angle 9°. For a 240 image scan parameters were as follows: acquisition matrix 64 × 64; field of view 220 × 220 mm; thickness 4.0 mm; gap 1.2 mm; repetition time 2,000 ms; echo time 30 ms; flip angle. 90°, 29 axial.

### 2.3 Data processing for fMRI

MRIcro software (Nottingham University, Nottingham, UK) was used to classify functional data, and to identify and exclude incomplete or flawed data. Resting-state functional MRI data were preprocessed using SPM8 (*Wellcome Department of Imaging Neuroscience, London, UK*) (https://www.fil.ion.ucl.ac.uk/spm/) and the REST software (*REST, V1.8*), (http://www.restfmri.net/forum/). Briefly, preprocessing included motion correction, spatial normalization, and spatial smoothing. The remaining data were processed using DPARSFA (*V2.3*) (*10 volumes*) (http://rfmri.org/DPARSF), including space standardization, head movement correction, slice time, digital image format conversion, and smoothing with a Gaussian kernel of 6 × 6 × 6 mm^3^ full width at half height. To obtain a balance among signals and to make the participants adapt to the noise generated during scanning, under normal circumstances, the first 10 volumes of functional magnetic resonance images were regarded as invalid data and were excluded. Data from participants whose maximum displacement in x, y, or z dimensions exceeded 1.5 mm or whose motion angle exceeded 15° were excluded. Linear regression was used in the present study to eliminate the influence of factors such as signals originating from white matter.

### 2.4 Statistical analysis

SPSS software, version 20.0 (IBM Corp., Armonk, NY, USA) was used to conduct independent sample t tests. *p* values less than 0.05 were considered statistically significant. Paired t-tests were carried out using REST software to analyze the functional data. Gaussian random field theory was used for multiple comparison correction, and the voxel level threshold was *p* < 0.005. AlphaSim, part of the REST toolbox, was used for correction at a cluster size >73 voxels and a level of *p* < 0.05. Receiver operating characteristic (ROC) curves provided a measure of sensitivity of mean ALFF values from brain regions of the patients and the healthy control group.

### 2.5 Clinical features correlation analysis

Different ALFF values in brain areas in AMD groups were categorized into regions of interest with rs-fMRI, and the relationship between the mean ALFF value in different brain areas and clinical performance was assessed by using correlation analysis (*p* < 0.05 significant differences).

## 3 Results

### 3.1 Demographics and behavioral results

AMD patients and HCs were aged 55.25 ± 4.04 years and 53.87 ± 5.16 respectively, and disease duration was 3.34 ± 2.88 months. The groups were statistically similar in age (*p* = 0.785) and weight (*p* = 0.542), but were statistically different in terms of best-corrected visual acuity (VA) of the left and right eyes (*p* = 0.004 and *p* = 0.003 respectively) (As described in the previous study (33), Supplementary Table S1).

### 3.2 ALFF differences

**Figure 2:**
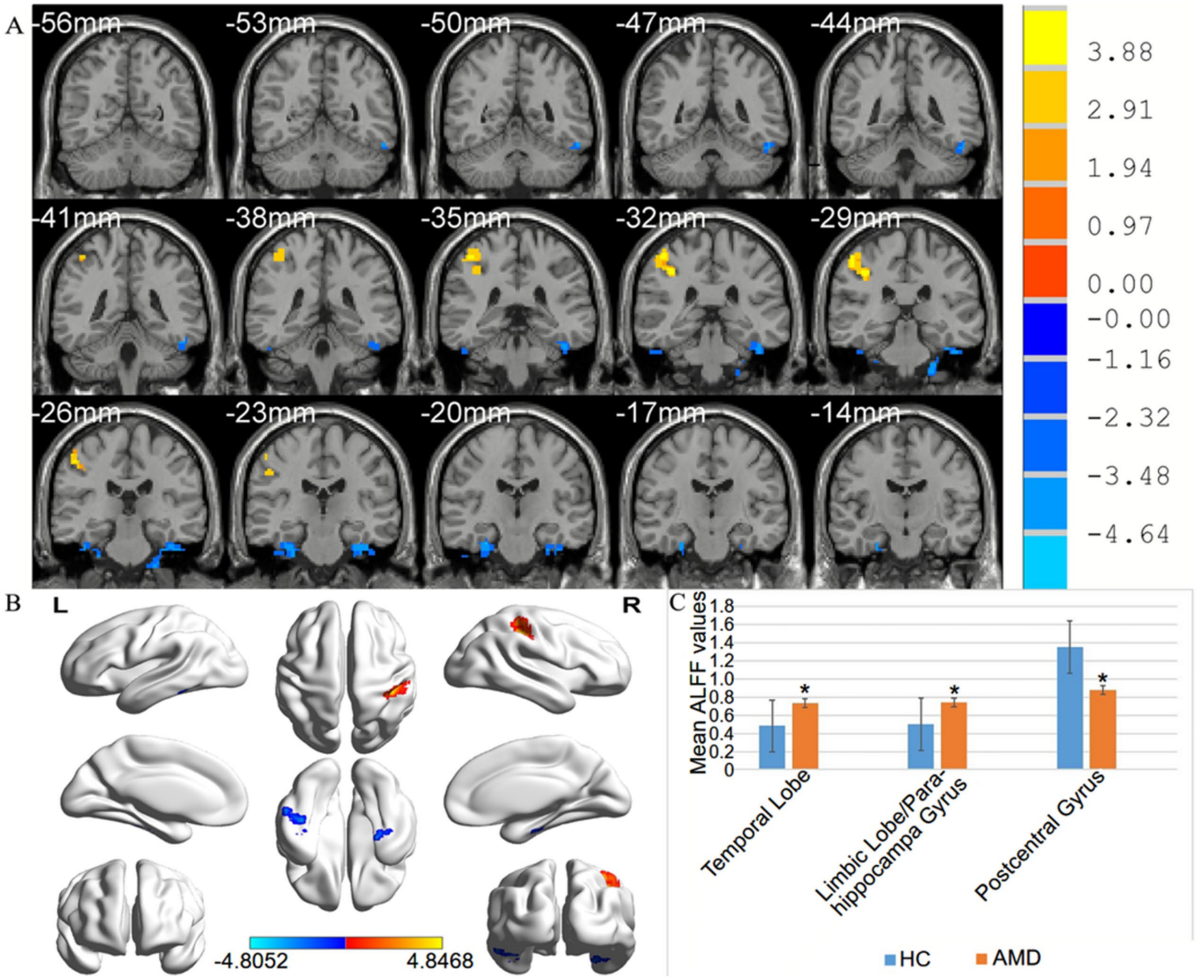
**(A,B)** Spontaneous brain activity in the AMD patients and the HC group. **(C)** Histogram of mean ALFF values in AMD and HCs groups. Warmer shades (yellow and red) represent moderate and high signal strength, respectively and blue represents lower signal strength. The signal value of the postcentral gyrus in AMD patients is lower than in controls, and on the contrary, the signal valuse of limbic lobe/ parahippocampa gyrus and temporal lobe are higher than controls. HCs, healthy controls; ALFF, the amplitude of low-frequency fluction; AMD, age-related macular degeneration.

From Figure 2 and Table 1, we can see that in comparison with HCs, the ALFF values of AMD patients were significantly higher in the temporal lobe, parahippocampal gyrus/limbic lobe, but significantly lower in the postcentral gyrus (Figure 2 and Table 2).

**Table 1:** ALFF method applied in opthalmological diseases.

Author	Year	Disease
Li et al. (1)	2016	Blindness
Hu et al. (15)	2021	Hypertensive retinopathy
Wang et al. (29)	2022	Strabismic amblyopia
Tan et al. (30)	2016	Unilateral acute open globe injury
Kang et al. (31)	2019	Acute eye pain
Shi et al. (32)	2019	Corneal ulcer

**Table 2:** Brain regions where ALFF values differ significantly between AMD patients and HCs.

Brain areas	MNI coordinates			Number of voxel	*T* value
	X	Y	Z		
HC < AMD					
Temporal Lobe	−24	−30	−51	146	−4.398
Limbic lobe/ Parahippocampa Gyrus	30	−21	−30	79	−4.8052
HC > AMD					
Postcentral Gyrus	39	−33	42	123	4.8468

The statistical threshold was set at voxel with *p* < 0.05 for multiple comparisons using GRF theory (*z* > 73, cluster-wise *p* < 0.05 corrected).

ALFF, the amplitude of low-frequency fluction; AMD, age-related macular degeneration; MNI, Montreal Neurological Institute.

### 3.3 Analysis of ROC curves

**Figure 3:**
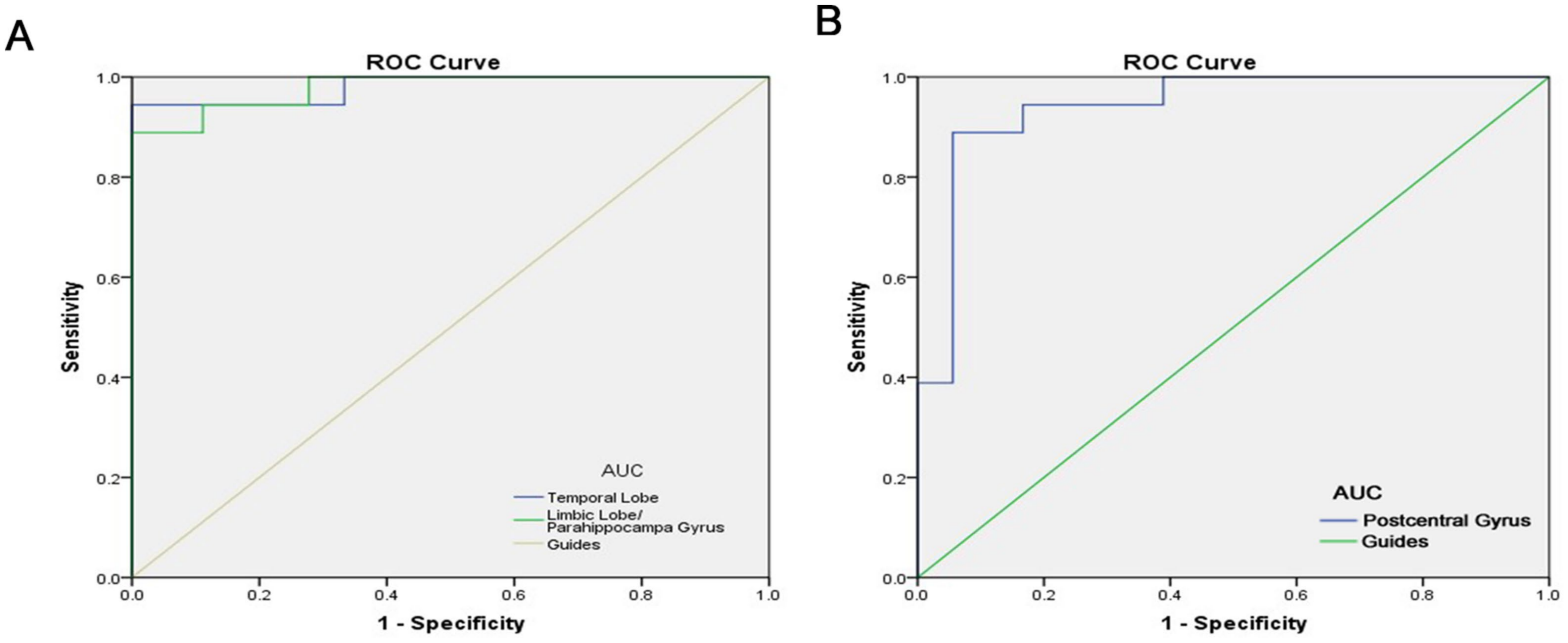
ROC curve analysis of the mean ALFF difference for altered brain regions. **(A)** The area under the ROC curve were 0.981, (*p* < 0.0001; 95% CI: 0.942–1.000) for Temporal Lobe, Limbic Lobe/ Parahippocampa Gyrus 0.978, (*p* < 0.0001; 95% CI: 0.940–1.000). **(B)** The area under the ROC curve were 0.941, (*p* < 0.0001; 95% CI: 0.863–1.000) for Postcentral Gyrus. AUC, area under the curve; ROC, receiver operating characteristic.

The under the curve of the ROC (AUC) provides an indication of diagnostic accuracy. AUC ranges from 0 to 1, higher values indicating higher accuracy. From Figure 3, AUC for the temporal lobe and the limbic lobe/parahippocampal gyrus were 0.981 and 0.978, respectively, (*p* < 0.0001). The AUC of the postcentral gyrus was also high, at 0.941 (*p*<0.0001), indicating that ALFF is a reliable marker for differentiating between the two groups. The high diagnostic accuracy of ALFF suggests its potential utility in the early detection of AMD. However, further studies with larger sample sizes are needed to confirm these findings and assess the clinical utility of ALFF in conjunction with other diagnostic tools such as OCT imaging or visual acuity tests.

### 3.4 Correlation analysis

**Figure 4:**
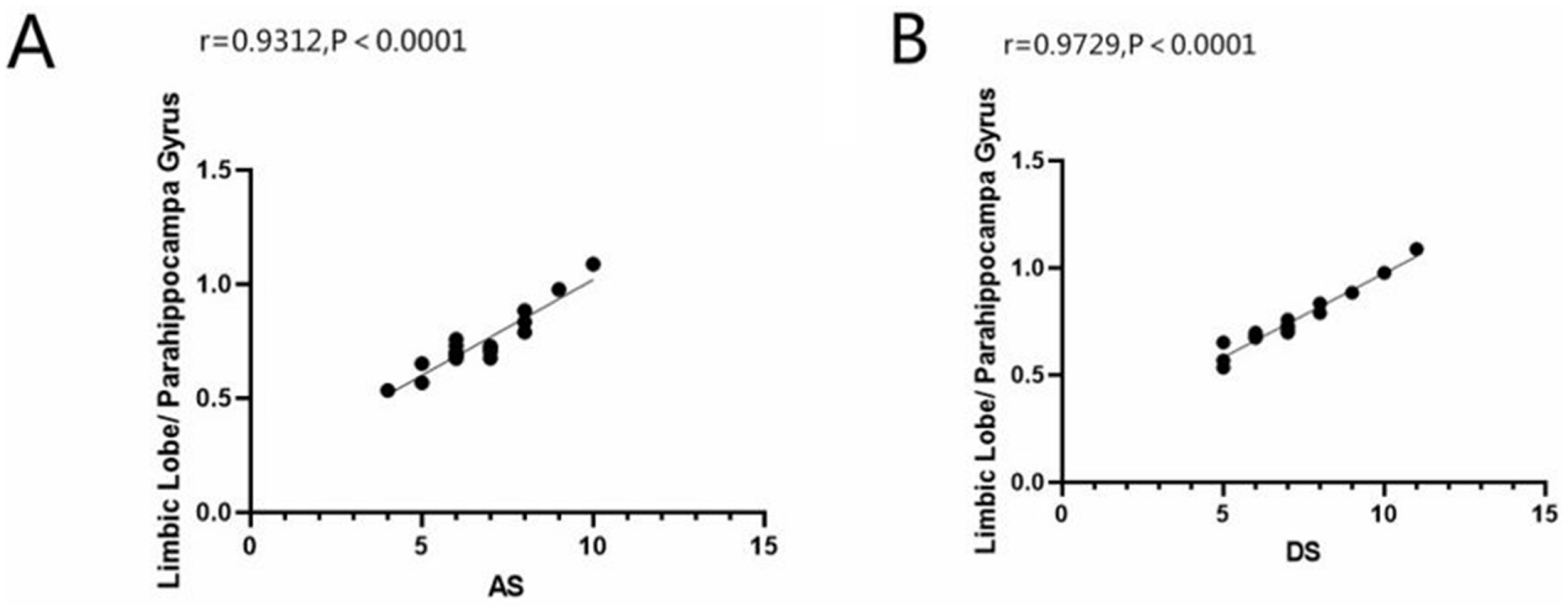
Correlation between the ALFF value of limbic lobe/ parahippocampa gyrus with AS and DS. In the AMD group, we founded that the ALFF value of limbic lobe/ parahippocampa gyrus showed a positive correlation with the anxiety scores **(A)** (*r* = 0.9312, *p*<0.0001) and the depressed scores **(B)** (*r* = 0.9729, *p*<0.0001). ALFF, the amplitude of low-frequency fluction; AS, anxiety scores; DS, depressed scores; AMD, age-related macular degeneration.

We can draw the conclusion from Figure 4, ALFF values of the limbic lobe/ parahippocampal gyrus were positively correlated with anxiety scores (*r* = 0.9312, *p* < 0.0001) and depression scores (*r* = 0.9729, *p* < 0.0001) in the AMD group.

## 4 Discussion

**Figure 5:**
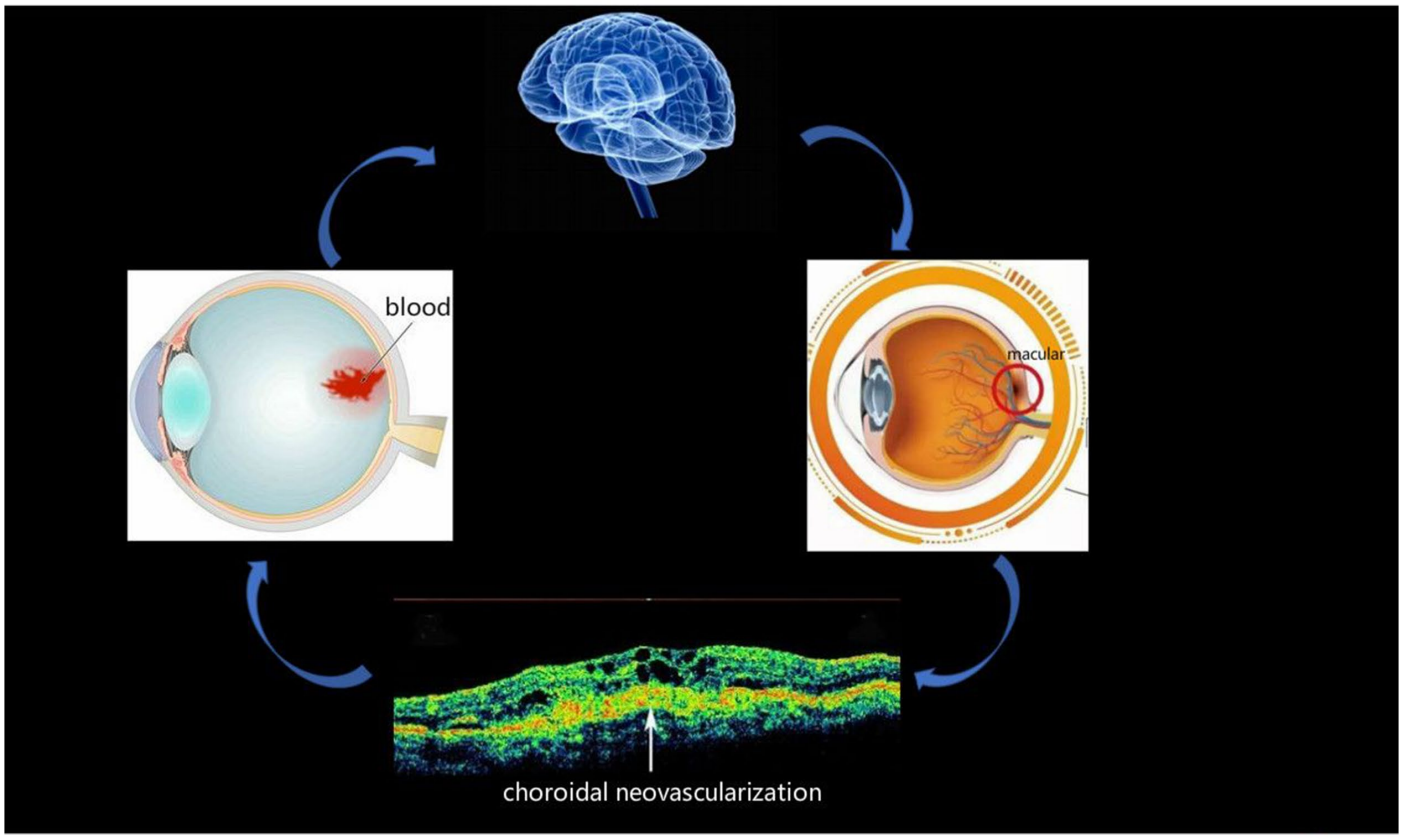
Relationship between images and clinical performances in AMD. macular degeneration may lead to the formation of choroidal neovascularization, and there is a possibility of bleeding from the fundus and brain activities. AMD, age-related macular degeneration.

In this study, we used the ALFF method to evaluate the spontaneous brain activity of AMD patients at rest, and the technology is widely used to study a variety of eye conditions, such as blindness (1), hypertensive retinopathy (15), strabismic amblyopia (29), unilateral acute open globe injury (30), acute eye pain (31), and corneal ulcer (Table 1) (32). At present, the incidence of AMD is increasing, and it is the main cause of blindness among the elderly, so it is worthy of attention. To date, research on this disease has focused on its clinical manifestations, pathogenesis, and treatment. The present study attempts to increase understanding of the disease by recording brain activity in AMD patients. The pathogenesis of AMD, which has received attention, usually leads to choroidal neovascularization, and this may cause fundal bleeding and even altered brain activity (Figure 5). The latter suggests that ALFF recordings may be useful in early diagnosis method for AMD.

**Figure 6:**
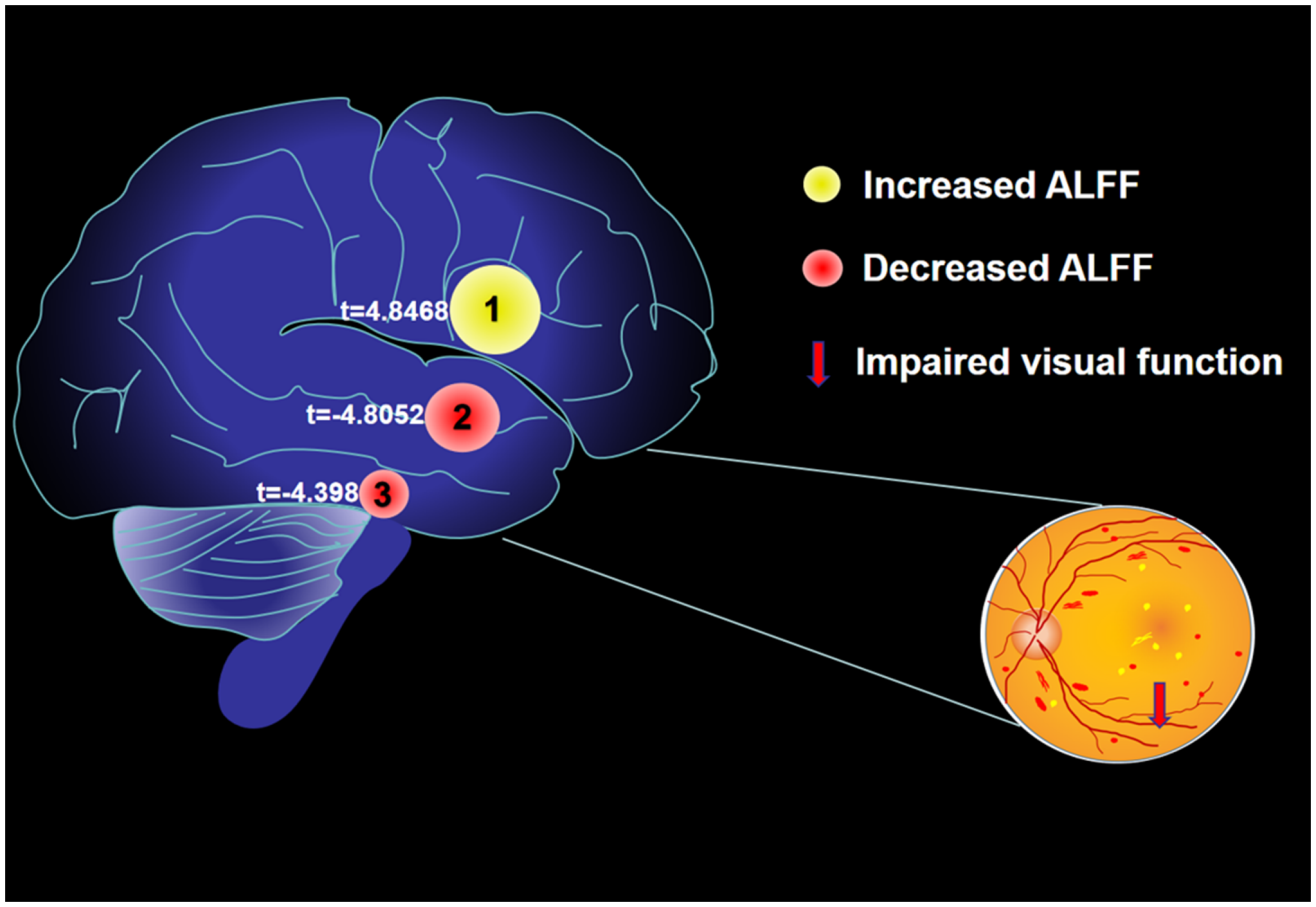
The mean ALFF values of altered brain regions. Compared with the HCs, the ALFF values of the following regions were decreased to various extents: 1- Postcentral Gyrus (*t* = 4.8468). Compared with the HCs, the ALFF values of the following regions were increased to various extents: 2- Limbic Lobe/ Parahippocampa Gyrus (*t* = −4.8052), 3- Temporal Lobe (*t* = −4.398). HCs, healthy controls; BA, Brodmann’s area.

In our previous research, we observed different fMRI parameters in different studies. In previous study, the regional homogeneity (ReHo) method was used to investigate levels of cerebral homogeneity in individuals with AMD. Mean ReHo values at the cingulate gyrus and the superior frontal gyrus were negatively correlated with clinical symptoms (33). The present research results show that compared with HC, AMD patients have higher ALFF values in the temporal lobe/parahippocampal gyrus, and limbic lobe, and lower ALFF values in the postcentral gyrus area (Figure 6). The higher ALFF values observed in AMD patients could be a result of compensatory neural activity in response to visual loss. As the brain reorganizes due to sensory deprivation, areas traditionally involved in visual processing, such as the occipital cortex, may exhibit increased activity. This phenomenon has been observed in other forms of sensory loss and may reflect heightened neural recruitment in regions involved in sensory processing and cognitive compensation (34–36). The decreased ALFF values observed in the postcentral gyrus could reflect cortical reorganization associated with sensory deprivation. As patients with AMD experience visual loss, compensatory mechanisms might lead to a decrease in sensory processing in the somatosensory cortex, or alterations in the cross-modal plasticity between sensory areas may contribute to this effect (37). Further investigation is needed to confirm these mechanisms. The increased ALFF values observed in the temporal lobe and limbic system may reflect compensatory neural activity linked to cortical reorganization due to visual loss. Cross-modal plasticity is a well-established phenomenon where the brain adapts to sensory deprivation by enhancing neural activity in non-visual areas, such as the temporal lobe (38). Additionally, the limbic system, which is involved in emotional processing, might undergo functional alterations in response to the emotional and cognitive impacts of AMD, including anxiety, depression, or cognitive decline (39). This could contribute to the observed increased ALFF values in these regions.

The temporal lobe is a complex brain area and is involved in various cognitive functions, such as language, memory, hearing, semantic processing, and vision (40). Previous research suggests that the temporal lobe has a role in epilepsy and autism (41), though these studies may have been affected by selective bias. One study found that picture naming triggered activation of the left posterior and inferior parts of the temporal lobe, suggesting that connected activity of parts of the temporal lobe was related to better naming performance (42). The posterior middle temporal gyrus is thought to represent a key connection between initial speech processing and subsequent semantic processing (43). Vaz et al. (44) reported that the process of episodic memory retrieval involves connection between the medial temporal lobe memory system and neocortex, and this connection may be the basis of memory retrieval. This is consistent with findings that the medial temporal lobe plays a vital role in human intermittent memory (32, 45, 46). Together with studies showing that the temporal lobe may be related to facial recognition (47), this body of research highlights the role of the temporal lobe in multiple functions. The present study found that temporal lobe ALFF values are higher in patients with AMD than in controls, indicating that these functions may be abnormal in AMD (Table 3).

**Table 3:** Different brain areas and their potential effects.

Brain areas	Experimental results	Brain function	Expected results
Temporal lobe	AMD>HCs	Cognitive function, language, memory, hearing, semantic processing, vision	Cognitive dysfunction
Limbic lobe/ Parahippocampa gyrus	AMD>HCs	Emotion, memory, movement, cognitive function, smell	Emotional problems, memory disorders and limited cognition
Postcentral gyrus	AMD<HCs	Sensation, pain, vibration and nociception	Sensory disturbance

AMD, age-related macular generation; HCs, healthy controls.

The parahippocampal gyrus is the core part of the limbic system, which plays a significant part in emotion and memory (48), especially visual memory (49), but not familiarity (50). Karanian et al. (51) found that false memory generated more activity in the parahippocampal cortex than true memory, suggesting that this area mediates context rather than sensory processing. Other research has shown that parahippocampus activity is related to negative emotions (52). In the present study, the ALFF value at the parahippocampal gyrus was higher in AMD patients than in controls, which may indicate overactivation of the limbic system, possibly accompanied by negative emotion.

In addition to emotion, the limbic system processes sensory input from the internal and external environment related to movement and cognitive functions (53). Because the limbic lobe is involved in the sense of smell and related functions, it is also called the rhinencephalon, or olfactory brain (54). Higher ALFF values of the limbic lobe in AMD may be accompanied by changes in smell, cognition, and other functions.

Finally, in the present study ALFF values at the postcentral gyrus are reduced in AMD patients compared with controls, which is contrary to previous observations, the possible reasons are the different sample sizes between this study and previous studies, as well as the factors affecting the experimental results of the subject’s own conditions. The postcentral gyrus is located in the main somatosensory cortex (S1) and plays an important role in sensation (55). A functional imaging study showed that the S1 activity observed during voluntary movement of the limbs is related to the activity of the movement area, and is not triggered by passive movement (56). Functional imaging has also shown that hemiplegic cerebral palsy is related to abnormal processing in the ipsilateral primitive somatosensory cortex (57). Studies have proposed that the S1 cortex is important in the location and discrimination of pain (58), and the plasticity of S1 plays a role in neuropathic pain (59). Lenoir et al. (60) found that S1 is involved in sensing vibration and in nociception. Together with these findings, the observation of low postcentral gyrus ALFF values in AMD patients in the present study suggests that these patients have limited function related to central posterior gyrus functional activity.

In addition to the above results, we also conducted a comparative study on the emotional-related clinical manifestations of AMD patients. The results show that in limbic lobe/ parahippocampa gyrus, the ALFF value is positively correlated with the AS and DS scores, which may indicate spontaneously hyperactive brain regions may be functionally active and cause emotional problems such as anxiety and depressed. Therefore, we can assume that areas with low spontaneous activity in the brain may trigger the opposite behavior.

Despite the AMD findings were explored in this study, there are some limitations. The ROC curve analysis demonstrated promising results, with a high AUC value, indicating the potential of ALFF as a diagnostic biomarker for AMD. However, these findings should be interpreted cautiously given the relatively small sample size, and further validation in larger cohorts is necessary. Future studies could also explore the combined use of ALFF with other clinical biomarkers, such as visual acuity or OCT, to enhance diagnostic precision and improve clinical outcomes. Meanwhile, potential confounding factors, such as AMD somatotype, could influence ALFF results. Future studies should stratify AMD patients into subgroups based on these factors to explore their effect on brain activity and better understand the underlying mechanisms of altered ALFF in AMD. We recognize that the relatively small sample size in this study may limit the statistical power and generalizability of the findings. Future studies with larger, multicenter cohorts are needed to validate these results and explore the broader applicability of ALFF as a diagnostic marker for AMD.

In conclusion, our study demonstrates that ALFF alterations in specific brain regions, including the occipital cortex, temporal lobe, and limbic system, could serve as potential biomarkers for the early detection of age-related macular degeneration (AMD). These findings suggest that changes in spontaneous brain activity may reflect neural adaptations to sensory deprivation. Future studies should explore the longitudinal dynamics of ALFF changes as AMD progresses, and investigate the potential of ALFF in combination with other diagnostic tools, such as OCT and visual acuity assessments, to enhance early diagnosis and therapeutic monitoring.

## Data Availability

The datasets used and analyzed during the current study are available corresponding author on reasonable request. The corresponding author should be contacted if someone wants to request the data from this study.
