# Supplementary material for: The amplitude of low frequency fluctuation and spontaneous brain activity alterations in age-related macular degeneration
Source: Front Med (Lausanne). 2025 Jan 22;11:1507971. doi: 10.3389/fmed.2024.1507971 (PMC11794247; doi:10.3389/fmed.2024.1507971)
Supplement: Supplementary file 1 [file Table_1.docx]

**Supplementary table 1.** Demographics and Clinical Measurements of AMD and HC Groups.

| Condition | AMD | HC | t | P-value* |
| --- | --- | --- | --- | --- |
| Male/female | 10/8 | 10/8 | N/A | >0.99 |
| Age (years) | 55.25±4.04 | 53.87±5.16 | 0.375 | 0.785 |
| Weight (kg) | 61.58±11.84 | 69.36±12.78 | 0.542 | 0.542 |
| Handedness | 18R | 18R | N/A | >0.99 |
| Best-corrected VA-L | 0.15±0.10 | 1.05±0.10 | -4.836 | 0.004 |
| Best-corrected VA-R | 0.10±0.05 | 1.05±0.15 | -4.736 | 0.003 |
| Duration of AMD (months) | 3.34±2.88 | N/A | N/A | N/A |

| IOP-L | 12.14±3.64 | 14.36±3.76 | 0.312 | 0.898 |
| --- | --- | --- | --- | --- |
| IOP-R | 14.26±3.97 | 15.95±4.12 | 0.336 | 0.802 |

**Note:** Independent t-tests comparing the two groups (p<0.05 represented statistically signiﬁcant differences). Data shown as mean standard deviation or n.

**Abbreviations:** AMD, age-related macular degeneration; HC, healthy control; L, left; R, right; N/A, not applicable; VA, visual acuity; IOP, intraocular pressure.
